# The influences of ammonia on aerosol formation in the ozonolysis of styrene: roles of Criegee intermediate reactions

**DOI:** 10.1098/rsos.172171

**Published:** 2018-05-02

**Authors:** Qiao Ma, Xiaoxiao Lin, Chengqiang Yang, Bo Long, Yanbo Gai, Weijun Zhang

**Affiliations:** 1 Laboratory of Atmospheric Physico-Chemistry, Anhui Institute of Optics and Fine Mechanics, Chinese Academy of Sciences, Hefei 230031, People's Republic of China; 2 University of Science and Technology of China, Hefei 230026, People's Republic of China; 3 School of Environmental Science and Optoelectronic Technology, University of Science and Technology of China, Hefei 230026, People's Republic of China; 4 School of Materials Science and Engineering, Guizhou Minzu University, Guiyang 550025, People's Republic of China

**Keywords:** ozonolysis, ammonia, Criegee intermediate, secondary ozonide

## Abstract

The influences of ammonia (NH_3_) on secondary organic aerosol (SOA) formation from ozonolysis of styrene have been investigated using chamber experiments and quantum chemical calculations. With the value of [O_3_]_0_/[styrene]_0_ ratios between 2 and 4, chamber experiments were carried out without NH_3_ or under different [NH_3_]/[styrene]_0_ ratios. The chamber experiments reveal that the addition of NH_3_ led to significant decrease of SOA yield. The overall SOA yield decreased with the [NH_3_]_0_/[styrene]_0_ increasing. In addition, the addition of NH_3_ at the beginning of the reaction or several hours after the reaction occurs had obviously different influence on the yield of SOA. Gas phase reactions of Criegee intermediates (CIs) with aldehydes and NH_3_ were studied in detail by theoretical methods to probe into the mechanisms behind these phenomena. The calculated results showed that 3,5-diphenyl-1,2,4-trioxolane, a secondary ozonide formed through the reactions of C_6_H_5_ĊHOO· with C_6_H_5_CHO, could make important contribution to the aerosol composition. The addition of excess NH_3_ may compete with aldehydes, decreasing the secondary ozonide yield to some extent and thus affect the SOA formation.

## 1. Introduction

Styrene is a highly reactive alkene with typical abundances ranging from 0.06 to 45 ppb in ambient atmosphere [1–3]. It can be emitted into atmosphere from abundant anthropogenic sources, such as adhesives, solvents, tobacco smoke and automobile exhausts [4,5]. With potential carcinogenic and mutagenic characteristics, styrene is known to be toxic to humans, and it can cause damage to the central nervous and reproductive systems if exposure to it occurs [6]. Furthermore, styrene is susceptible to reaction with ozone to form secondary organic aerosol (SOA), resulting in secondary pollution in the atmosphere [7].

Criegee mechanism, first proposed by Rudolf Criegee in 1949 [8], is widely accepted in the ozonolysis of unsaturated hydrocarbons in atmospheric chemistry. Criegee intermediates (CIs) are formed by the ring-opening reaction of a rather unstable primary ozonide (POZ) which formed directly by the 1,3-cycloaddition of O_3_ across the double bond. Because of short lifespan and unavailable direct precursor, experimental studies about CI have not been carried out until recent years. Taatjes *et al.* first observed the simplest CI (·CH_2_OO·) directly using the tunable synchrotron photoionization combined with multiplexed mass spectrometry [9]. Thereafter several studies have been carried out to detect ·CH_2_OO· and (CH_3_)_2_ĊOO· and study the kinetics of their unimolecular reactions using synchrotron photoionization mass spectrometry and spectroscopic methods [10–14]. More researches have been done using theoretical methods to investigate the reaction kinetics and mechanisms of bimolecular reactions, including the reactions of CI with NH_3_, NO_x_, SO_2_, (H_2_O)_x_, HO_x_ and others [7,15–26].

In addition of CI, aldehydes are also formed as co-products in the decomposition of POZ and can recombine with CI generating the more stable secondary ozonide (SOZ) intermediate. In the past few years, the SOZs were detected by several researches in the gas phase ozonolysis of simple alkenes [27–29]. At 730 Torr, a SOZ propene ozonide (methyl-1,2,4-trioxolane) was observed as the major product in the reaction of ·CH_2_OO· with CH_3_CHO, indicating collisional stabilization of the nascent SOZ near atmospheric pressure [30]. Analogously, Tuazon *et al.* proposed that C_6_H_5_ĊHOO· can combine with C_6_H_5_CHO to form a SOZ structure (3,5-diphenyl-1,2,4-trioxolane, DPSOZ) in the ozonolysis of styrene [2]. And it may undergo partial conversion into a hydroxyl-substituted ester (C_6_H_5_CH(OH)OC(O)C_6_H_5_). This secondary ozonide has very low vapour pressure, which makes it easy to partition into the aerosol phase from gas phase and contribute a major composition in styrene-ozone oxidation reactions [7]. In addition, Winterhalter *et al.* [31] and Nguyen *et al.* [32] also verified the formation of internal SOZ in the reaction of β-caryophyllene with O_3_ experimentally and theoretically, respectively. Therefore, in the ozonolysis of alkenes, reactions of CI with the simultaneously generated aldehydes in the system should be further studied because they may have an important influence on the distribution of products in both the gas phase and particle phase.

Jalan *et al.* studied the reaction mechanisms and kinetics for reactions of ·CH_2_OO· with HCHO, CH_3_CHO and CH_3_COCH_3_, in which SOZ and organic acids were formed, and the tendency to form SOZ was proposed in the order HCHO < CH_3_CHO < CH_3_COCH_3_ [33]. Recently, Wei *et al.* investigated the detailed potential energy surface (PES) for the reaction of ·CH_2_OO· with CH_3_CHO, and proposed a slightly different pathway of SOZ isomerization [34]. All these theoretical calculations are only for the reactions between aldehydes with the simplest structures. Thus, it is of great necessity for investigating CIs reaction with aldehydes to understand the formation of SOAs in the ozonolysis of alkenes.

Furthermore, ammonia (NH_3_) is an important alkaline constituent and plays an important role in the atmosphere [35,36]. Atmospheric NH_3_ is emitted by different biogenic and anthropogenic sources, such as soil, vegetation, livestock waste, NH_3_-based fertilizer volatiles, mobile exhaust and biomass combustion emissions [35–38]. In addition to reactions with sulfuric acid and nitric acid to form ammonium sulfate and ammonium nitrate aerosols [39], NH_3_ also participates in atmospheric oxidation process. For styrene-ozone system, the presence of ammonia decrease the SOA yield [7]. The authors considered that the major condensable species for this system are 3,5-diphenyl-1,2,3-trioxolane and a hydroxyl-substituted ester, and hypothesize that the presence of NH_3_ may attack these two products causing their rapid decomposition. While for α-pinene ozonolysis reactions, SOA yield increased when NH_3_ was added after the reaction ceased [21]. The resulting aerosol growth may be attributed to ammonium salts formed by the reaction between organic acids and NH_3_. Recently, Liu *et al*. investigated the influence of NH_3_ on particle formation from gasoline vehicles exhausts [40]. Adding NH_3_ into the reactor after 3 h photo-oxidation of these complex mixtures, the particle number concentration and mass concentrations increased rapidly, but the average carbon oxidation state of SOA remained almost unchanged. In theoretical calculations, Jørgensen & Gross investigated the reactions between NH_3_ with a secondary ozonide and a hydroxyl substituted ester firstly, which formed in the ozonolysis of ethene [22]. Then the reactions between NH_3_ and three simplest carbonyl oxides (H_2_COO, CH_3_HCOO and (CH_3_)_2_COO) were studied detail [23]. The estimated reaction rates of carbonyl oxides and NH_3_ range from 1.8 × 10^−13^ to 5.1 × 10^−18^ cm^3^ molecule^−1^ s^−1^, which is several orders of magnitude higher than between secondary ozonide and NH_3_. It may indicate that the influence of NH_3_ on the formation of SOA is mainly by reacting with carbonyl oxides.

In this article, the ozonolysis of styrene was studied both experimentally and theoretically to explore the formation of SOA. Different from many researches investigating the contribution of NH_3_ to atmospheric nucleation [41,42], NH_3_ was added to investigate its influence on the formation of SOA in the reaction. As mentioned above, the reactions of CIs with aldehydes as well as NH_3_ in the system need to be considered. A series of experiments under different initial conditions were carried out in a Teflon chamber, while the mechanism and the PESs for reactions between CIs and aldehydes and NH_3_ involved were studied in detail by means of theoretical calculations. Finally, the influence of NH_3_ on the formation of SOA was discussed by comparing the competitive reactions of NH_3_ and aldehydes with CIs.

## 2. Material and methods

### 2.1. Experimental materials and methods

Experiments were carried out in a simulation chamber with the volume of the FEP-Teflon reactor about 6 m^3^ (1.7 m × 1.7 m × 2.1 m, surface/volume ∼ 3.30 m^−1^). The enclosure wall of the chamber is filled with thermally isolated material, and the inner side of the wall is covered by reflective stainless steel to achieve uniform light intensity. The reactor is fixed on top and bottom frames, and the top frame can be moved vertically, so the FEP reactor is collapsible. All the connection parts of the reactor are made of Teflon or stainless steel without O-rings in order to avoid the evaporation of volatile organic compounds into the reactor. To minimize the influence of wall effect, the sampling tubes are stretched to the middle of the reactor. Prior to each experiment, the reactor was cleaned by reducing the reactor volume to less than 10% of its original volume and refilling it to its maximum volume with purified air at least five times.

The AADCO pure air generator (Model 737, USA) is used to purify the ambient air, and then the purified air is used as the background and carrier gas. In order to further purify the air, compressed air from the AADCO generator is passed through two consecutive scrubbers filled with activated carbon and silica gel respectively. Residual hydrocarbons, NO, NO_2_, O_3_ (less than 1 ppbv), NH_3_ (less than 1 ppbv) and particles (less than 10 particles cm^−3^) are almost undetectable after passing the purification system. In a typical experiment, a known volume of the styrene (99%, Sigma-Aldrich) was injected into a temperature-controlled glass bulb vaporizer (maintained at around 373 K in this work) by micro syringe and flushed into the reactor by purified air. Ozone was generated by an adjustable ozone generator (COM-AD-01, ANSEROS, Germany). Ammonia standard gas (50 ppmv in N_2_) was provided by National Institute of Metrology, China, and added into the reactor through a mass flow controller. The temperature and relative humidity (RH) are monitored by a commercial temperature and humidity sensor (HC2-C05, Rotronic, China). The hydrocarbon concentration is detected by a gas chromatography equipped with flame ionization detector (GC-FID, Agilent Technologies, USA). O_3_ concentration is measured by an O_3_ analyser (Model 49i, Thermo Scientific, USA). NO, NO_2_, NO_x_ and NH_3_ mixing ratios are monitored using an NH_3_ analyser (Model 17i, Thermo Scientific, USA). A scanning mobility particle sizer (SMPS 3936, TSI, USA), which consists of a differential mobility analyser (DMA, TSI model 3080) and a condensation particle counter (CPC, TSI model 3775), is used to measure the SOA particle concentrations and size distribution as a function of the reaction time. All experiments were carried out at room temperature and dry conditions (RH < 5%).

### 2.2. Theoretical methods

The reactions between CI and aldehydes as well as NH_3_ involved in this work were studied using theoretical methods. Both the spin-unrestricted and spin-restricted form of the B3LYP functional was carried out on the transition states found in the reactions of CIs with formic acid in our previous study [24]. The results show that the UB3LYP and B3LYP energies, frequencies and geometrical parameters are identical. Therefore, all stationary points in the PESs were optimized using the B3LYP density functional method [43] and the 6-311G++(2d,2p) basis set. The harmonic vibrational frequencies were also calculated at the same level to characterize all stationary points as either minima or transition states. In addition, intrinsic reaction coordinate (IRC) calculations were performed for each transition state to confirm the connections between the expected reactants and products. The relative energies were obtained in high-level *ab initio* method CBS-QB3 [44,45]. In CBS-QB3 method, geometries are optimized and frequencies are calculated at the level of B3LYP/6-311G(d,p). Second, energy calculations at MP2/6-311+G(2df,2p) level are done and CBS extrapolation is calculated. Third, MP4(SDQ)/6-311G(d,p) and QCISD(T)/6-311G(d,p) single point energies are computed. Finally, two empirical correction terms: effect of absolute overlap integral and spin contamination, are considered in the overall energy estimate. All quantum chemical calculations in this work were performed with Gaussian 09 software package [46].

## 3. Results and discussion

### 3.1. Influence of NH_3_ on SOA yield

As listed in table 1, the experiments performed can be classified into three scenarios according to the corresponding experimental conditions. In group A, ozonolysis of styrene was studied under various [O_3_]_0_/[styrene]_0_ ratios without NH_3_. In group B and C, the effects of NH_3_ on SOA formation were studied. The difference is that, in group B, NH_3_ was added at the beginning of the reaction under different [NH_3_]_0_/[styrene]_0_ ratios, while in group C, excess NH_3_ was added after the reaction. All the experiments were carried out with the concentration of ozone in excess of styrene ([O_3_]_0_/[styrene]_0_ ratio range from 2 to 4), and styrene is consumed completely in each experiment. An average density of 1.2 g cm^−3^ was used to convert the total aerosol volume measured by DMA to total mass for aerosol formation from styrene ozonolysis.

**Table 1.** Initial conditions and results obtained from dark experiments.

expt. no.	HC_0_ (ppbv)	HC_0_(ΔHC) (μg m^−3^)	[O_3_]_0_ (ppbv)	[NH_3_] (ppbv)	[NH_3_]/HC_0_	*M*_0_ (μg m^−3^)	SOA yield (%)
A1	199	848	∼600	0	0	29	3.4
A2	277	1180	∼600	0	0	32	2.7
A3	467	1989	∼1100	0	0	157	7.9
A4	392	1670	∼1800	0	0	136	8.2
C1^a^	392	1670	∼1800	∼3000	∼8	109	6.5
A5	722	3076	∼2200	0	0	267	8.7
C2^a^	722	3076	∼2200	∼3000	∼4	196	6.4
A6	1314	5598	∼3500	0	0	456	8.1
C3^a^	1314	5598	∼3500	∼3000	∼2	367	6.6
B1	912	3885	∼2400	∼3000	∼3	120	3.1
B2	1469	6258	∼3500	∼3000	∼2	195	3.1
B3	378	1610	∼1100	∼3000	∼8	51	3.2
B4	930	3962	∼2000	800	∼0.86	169	4.3
B5	825	3515	∼1800	60	∼0.07	179	5.1
B6	715	3046	∼1800	20	∼0.03	212	7.0

^a^Data of this row and its adjacent row above are derived from the different stages of the same experiment.

According to the partitioning theory originally outlined by Pankow [47] and Odum *et al.* [48], SOA yield is defined as the ratio of the amount of SOA formed to the amount of hydrocarbon consumed,

3.1 $$Y=\frac{\Delta M_{0}}{\Delta \text{HC}},$$

where Δ*M*_0_ (μg m^−3^) is the mass of organic aerosol formed by the oxidation of ΔHC (μg m^−3^). The wall loss of volatile hydrocarbon is negligible for FEP-Teflon chamber, and the method of wall loss correction for aerosol has been described in our previous work by Hu *et al.* [49]. Briefly, particle wall loss can be described as

3.2 $$\frac{\text{d}N(d_{p})}{\text{d}t}=-k_{\mathrm{dep}}(d_{p})N(d_{p}),$$

where *N*(*d*_p_) is the concentration of particles and *k*_dep_(*d*_p_) is the deposition rate coefficient for particles with diameter *d*_p_ [50]. The relationship between *k*_dep_ and *d*_p_ can be determined by optimization of four parameters (*a*, *b*, *c* and *d*) with the experimental data [51].

3.3 $$k_{\mathrm{dep}}(d_{p})=ad_{p}^{b}+\frac{c}{d_{p}^{d}}.$$

Parameters *a*, *b*, *c* and *d* were optimized to be 0.0094, 0.60639, 1449.97666 and 2.45854 respectively for this chamber, and then *N*(*d*_p_) can be corrected and the suspended aerosol mass concentration Δ*M*_0_ can be calculated from the wall-loss corrected volume concentration.

**Figure 1.**
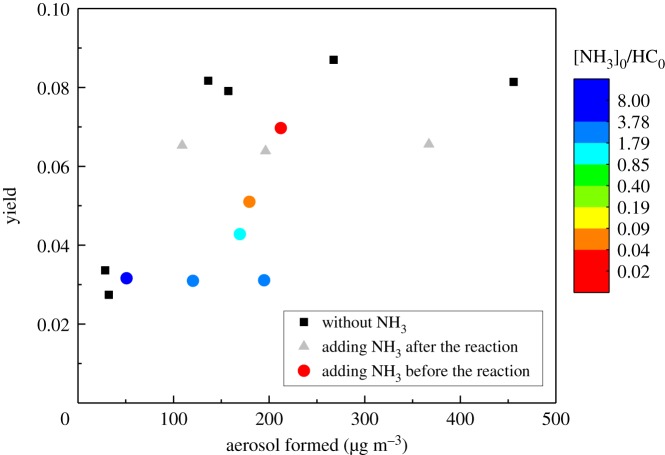
SOA yield from ozonolysis reactions of styrene without NH_3_ (black squares), with NH_3_ added after the reaction (grey triangles), and with NH_3_ added at the beginning of the reaction (coloured circles). The different colours in the circles represent different [NH_3_]_0_/[styrene]_0_ ratios.

After correcting with wall loss, the aerosol yields for all three group experiments are displayed in figure 1. Compared to the reactions without NH_3_, the addition of NH_3_ led to significant decrease in SOA yield. The different initial concentration of NH_3_ resulted in different SOA yield. Moreover, the addition of NH_3_ before and after the reaction had obviously different influence on the yield of SOA. When NH_3_ was added before the reaction (group B), the final SOA yield decreased significantly with the increase of [NH_3_]_0_/[styrene]_0_ ratio; while when NH_3_ was added after the reaction (group C), the yield of SOA was clearly higher than that under a similar [NH_3_]/[styrene]_0_ ratio in group B, but it was still lower than that without NH_3_ (group A).

**Figure 2.**
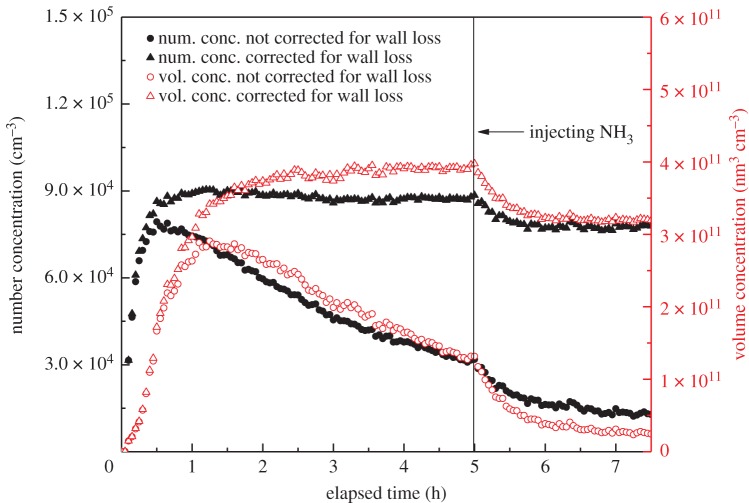
Changes in number and volume concentrations of SOA after the injection of NH_3_. (Experiment A6 and C3.)

Figure 2 shows the changes of SOA concentration in the reaction of styrene and ozone when NH_3_ was added 5 h after reaction starts. The solid black triangles and circles represent the number concentration of aerosol with and without wall loss correction, respectively. Similarly, the hollow red triangles and circles represent the volume concentration of aerosol with and without wall loss correction. As shown in figure 2, with NH_3_ added, both the number and volume concentrations of the SOA are decreased significantly. Na *et al.* also found an obvious decrease for the volume concentration of SOA when NH_3_ was added in the reaction of styrene with O_3_ [7], which was similar to our result. For the number concentration, it showed a slight increase in their work, which differs from the present result, probably because of the high excess ozone used in our experiment.

### 3.2. Competitive reactions of Criegee intermediate with aldehydes and NH_3_

As mentioned in the introduction, CIs and aldehydes generated in alkene ozonolysis reactions can further react with each other, producing a more stable SOZ which may have an important contribution to the aerosol phase. After the addition of NH_3_, the reactions between CIs and NH_3_ may compete with the reactions of CIs with aldehydes, which could affect the formation of SOA. In the following, the reactions of CIs with aldehydes and NH_3_ were studied in detail by theoretical methods to investigate whether the competition of these reactions affect the formation of SOA.

#### 3.2.1. Criegee intermediate reactions with aldehydes

**Figure 3.**
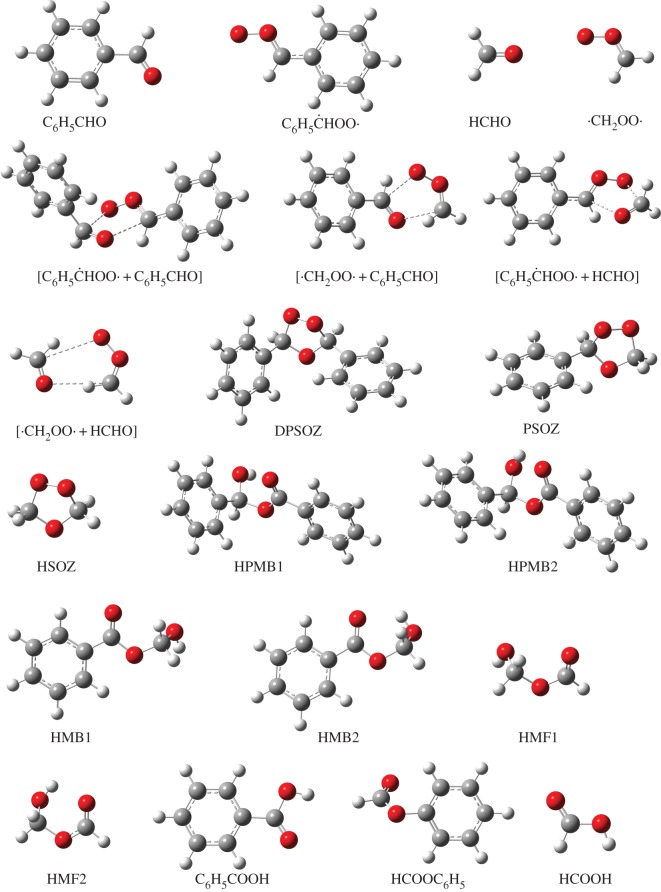
B3LYP/6-311G++(2d,2p)-computed structures of reactants, pre-reactive complex, intermediates and products in four different reactions of Criegee intermediate with aldehydes.

**Figure 4.**
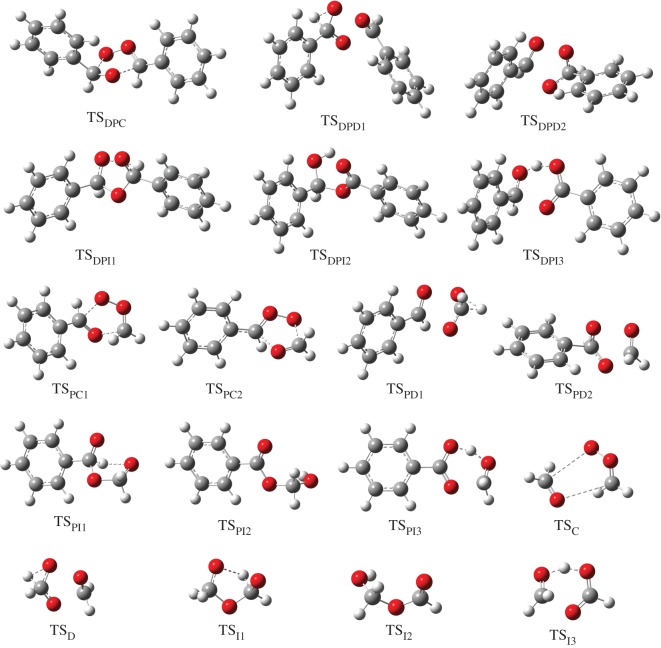
B3LYP/6-311G++(2d,2p)-computed structures of transition states in four different reactions of Criegee intermediate with aldehydes.

**Figure 5.**
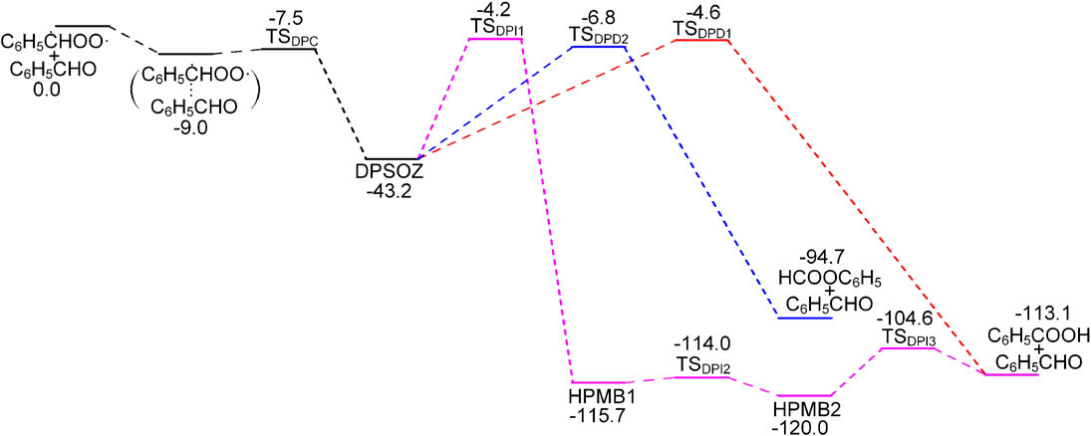
Potential energy surface (kcal mol^−1^) of the C_6_H_5_ĊHOO· + C_6_H_5_CHO reaction calculated at the CBS-QB3 level.

B3LYP/6-311G++(2d,2p) geometries for all the stationary points involved in reactions between the CIs and aldehydes are shown in figures 3 and 4. The PES for the C_6_H_5_ĊHOO· + C_6_H_5_CHO reaction calculated by CBS-QB3 is depicted in figure 5. Starting from separated C_6_H_5_ĊHOO· and C_6_H_5_CHO reactants, pre-reactive complex ([C_6_H_5_ĊHOO· + C_6_H_5_CHO]) is formed before the transition state. This leads to the formation of secondary ozonide (3,5-diphenyl-1,2,4-trioxolane, DPSOZ) by 1,3-cycloaddition (TS_DPC_) of C_6_H_5_ĊHOO· across the C=O bond in C_6_H_5_CHO. The reaction from C_6_H_5_ĊHOO· and C_6_H_5_CHO to DPSOZ is exothermic by 43.2 kcal mol^−1^.

There are three possible reaction paths for the isomerization and unimolecular decomposition of DPSOZ. The first reaction path is to form benzoic acid and benzaldehyde ([C_6_H_5_COOH + C_6_H_5_CHO]) via the transition state (TS_DPD1_) with a barrier of −4.6 kcal mol^−1^. TS_DPD1_ involves the break of the central O–O and C–O bond in DPSOZ accompanied by an H-shift process simultaneously. The second path is to generate phenyl formate (a product tentatively identified by Tuazon *et al.* [2]) and benzaldehyde ([HCOOC_6_H_5_ + C_6_H_5_CHO]) products via TS_DPD2_, which has the lowest energy saddle point (−6.8 kcal mol^−1^) and lies 2.2 kcal mol^−1^ below the TS_DPD1_. In this process, the O–O and the central C–O bond in DPSOZ break and one O atom shifts to the benzene ring simultaneously. The third isomerization pathway is more complicated, which involves three transition states and two intermediates (DPSOZ → TS_DPI1_ → HPMB1 → TS_DPI2_ → HPMB2 → TS_DPI3_). The reaction barrier of this process is 4.2 kcal mol^−1^ below the bimolecular reactants. Regardless of reaction barriers, C_6_H_5_CHO acts as a bridge for the isomerization of C_6_H_5_ĊHOO· to HCOOC_6_H_5_ and C_6_H_5_COOH.

**Figure 6.**
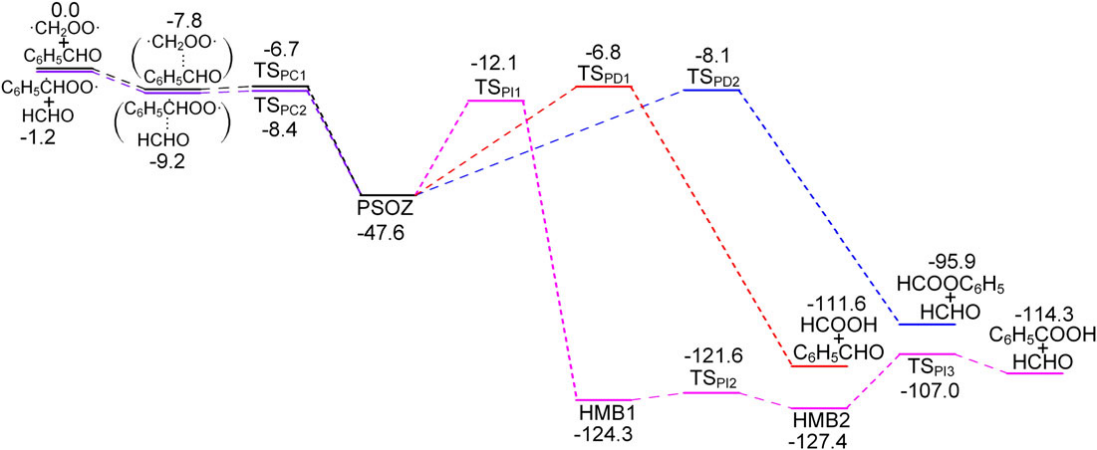
Potential energy surface (kcal mol^−1^) of the C_6_H_5_ĊHOO· + HCHO and ·CH_2_OO· + C_6_H_5_CHO reaction calculated at the CBS-QB3 level.

Both the reaction of C_6_H_5_ĊHOO· + HCHO and ·CH_2_OO· + C_6_H_5_CHO generate the same structure of secondary ozonide PSOZ (3-phenyl-1,2,4-trioxolane). The detailed reaction pathways are shown in figure 6. Both of them begin with the formation of a pre-reactive complex before the transition state. Similar to DPSOZ, the formation of PSOZ is highly exoergic and followed by decomposition under three different pathways. That is, (i) generate formic acid and benzaldehyde ([HCOOH + C_6_H_5_CHO]) through the transition state TS_PD1_; (ii) generate phenyl formate and formaldehyde ([HCOOC_6_H_5_ + HCHO]) through the transition state TS_PD2_; (iii) generate benzoic acid and formaldehyde ([C_6_H_5_COOH + HCHO]) through a more complicated pathway (PSOZ → TS_PI1_ → HMB1 → TS_PI2_ → HMB2 → TS_PI3_). It is worth noting that the energy of transition state TS_PI1_ is lower than TS_PD1_ by 5.3 kcal mol^−1^, indicating that the reaction pathway involving the reaction state TS_PI1_ is expected to make a major contribution.

**Figure 7.**
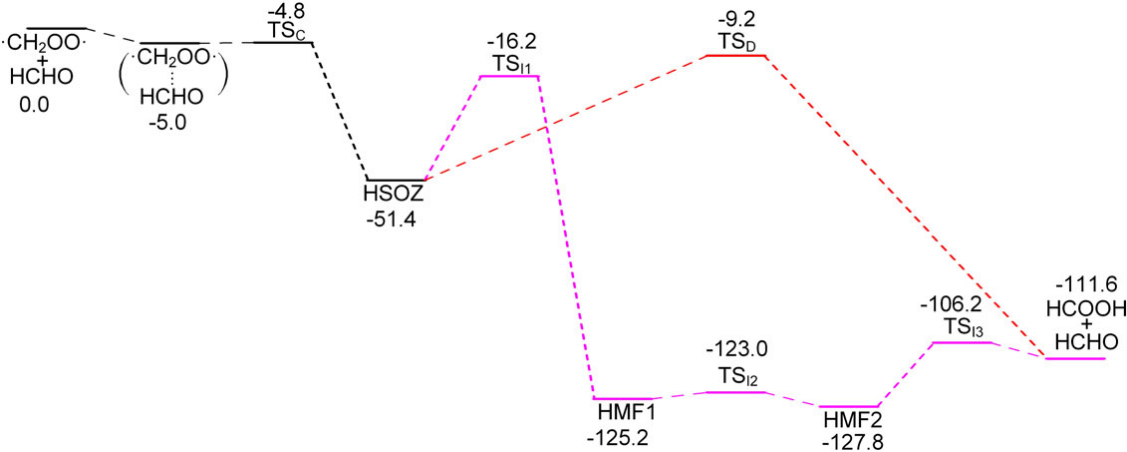
Potential energy surface (kcal mol^−1^) of the ·CH_2_OO· + HCHO reaction calculated at the CBS-QB3 level.

The reaction of ·CH_2_OO· + HCHO = HSOZ is 51.4 kcal mol^−1^ exothermic, according to CBS-QB3 theory, which is in good agreement with the result of 51.3 kcal mol^−1^ by the RCCSD(T)-F12a/VTZ-F12//B3LYP/MG3S approach (including zero point corrections) [33]. The profile of PES for this reaction is drawn in figure 7. The HSOZ decomposes into stable formic acid and formaldehyde ([HCOOH + HCHO]) through transition state TS_D_, which involves an H-shift process and the dissociation of the O–O and central C–O bond in HSOZ. This result is consistent with the recent conclusions of Jalan *et al.* [33]. An alternative isomerization pathway from HSOZ to the same products involved a series of transition states and intermediates (HSOZ → TS_I1_ → HMF1 → TS_I2_ → HMF2 → TS_I3_). However, our attempts at locating an optimized transition state structure connecting HSOZ and a 1,4-singlet biradical intermediate (abb. BIR in Jalan *et al.* [33]) were failed. Our calculation is consistent with a similar isomerization channel from 3-methyl-1,2,4-trioxolane to products by Wei *et al.* [34] (pathway A in the reference). The saddle point TS_I1_ is 7.0 kcal mol^−1^ lower than TS_D_ in energy, indicating that the latter pathway makes a major contribution in these competition channels.

#### 3.2.2. Criegee intermediate reactions with NH_3_

**Figure 8.**
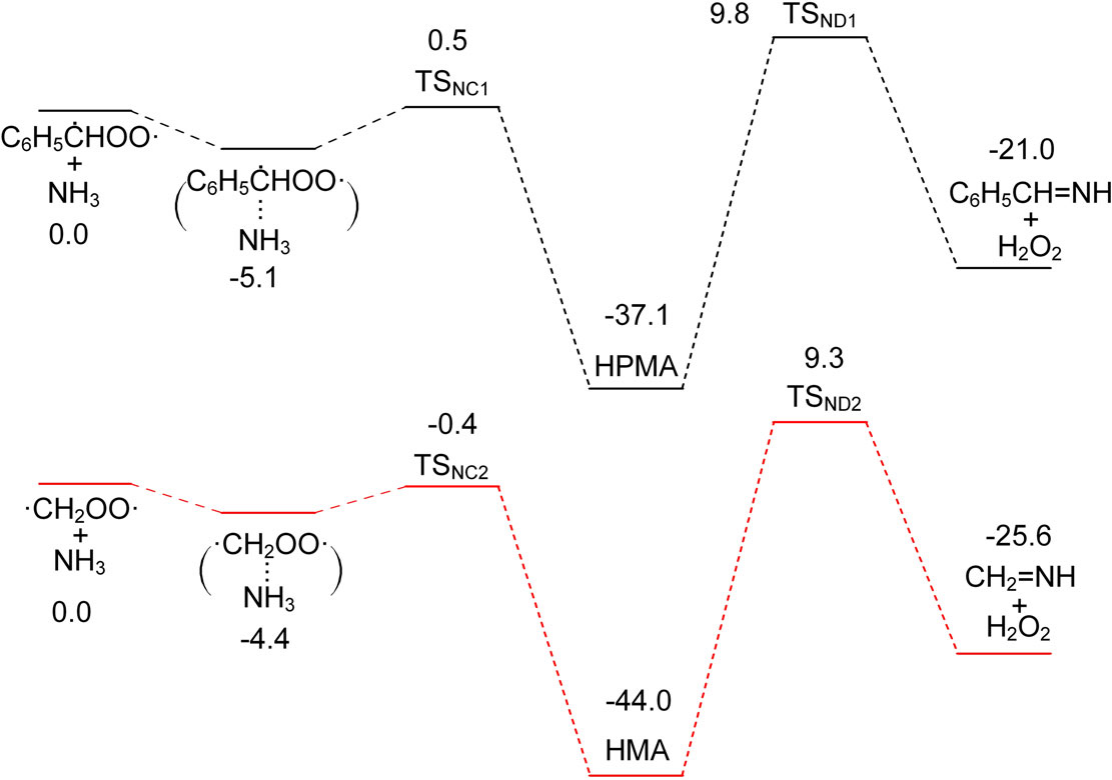
Potential energy surface (kcal mol^−1^) of C_6_H_5_ĊHOO· + NH_3_ and ·CH_2_OO· + NH_3_ calculated at the CBS-QB3 level.

**Figure 9.**
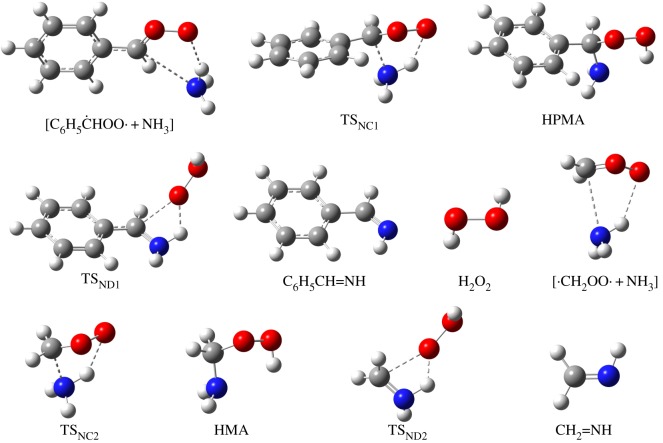
The structures of pre-reaction complexes, transition states, intermediates, and stabilized products in the reactions of C_6_H_5_ĊHOO· + NH_3_ and ·CH_2_OO· + NH_3_. All these structures are optimized at the B3LYP/6-311++G(2d,2p) level.

The profiles of PES for reactions of C_6_H_5_ĊHOO· and ·CH_2_OO· with NH_3_ are shown in figure 8. The structures of all the pre-reaction complexes, transition states, intermediates and stabilized products in the reactions are shown in figure 9. At the beginning of these reactions, there is a rapid pre-equilibrium between the reactants and the pre-reaction complex. Then an H atom in NH_3_ shifts to the terminal O atom in the COO moiety and the N atom and C atom form a new chemical bond. These isomerization reactions result in the formation of hydroperoxy(phenyl)methanamine (HPMA) and hydroperoxymethanamine (HMA) respectively. Afterwards, another H atom transfers from the N atom to O atom, accomplished by the break of C–O bond to generate phenylmethanimine (C_6_H_5_CH=NH) or methanimine (CH_2_=NH) with H_2_O_2_. For the reaction of C_6_H_5_ĊHOO· with NH_3_, the stabilization energy of the pre-reactive complex (*E*_stab_) is −5.1 kcal mol^−1^, the activation barrier (*E*_a_) is 5.6 kcal mol^−1^, and the reaction energy (Δ*E*_0_) is −37.1 kcal mol^−1^. And for the reaction of ·CH_2_OO· with NH_3_, the *E*_stab_, *E*_a_ and Δ*E*_0_ are −4.4 kcal mol^−1^, 4.0 kcal mol^−1^ and −44.0 kcal mol^−1^ respectively, which is consistent with the previous investigations performed with other different methods [23].

#### 3.2.3. Influence of Criegee intermediate reactions on SOA formation

From the discussion above, in the ozonolysis of styrene, the CIs (C_6_H_5_ĊHOO· and ·CH_2_OO·) could easily react with the simultaneously generated aldehydes (C_6_H_5_CHO and HCHO). The corresponding SOZ intermediate is formed through 1,3-cycloaddition of CI across the C=O bond which is a typical exothermal reaction without energy barrier. Although the isomerization of SOZ has a high energy barrier, the energy released by CI + aldehydes → SOZ may help it partly decompose. Formed without energy barrier and with low vapour pressure, DPSOZ (3,5-diphenyl-1,2,4-trioxolane) would make a major contribution to the aerosol composition. This conclusion is consistent with the previous experimental results [2,7]. However, unlike the previous conclusion that hydroxyl(phenyl)methyl benzoate (C_6_H_5_CH(OH)OC(O)C_6_H_5_) may be one of the products, our theoretical calculations show that these intermediates (HPMB1 and HPMB2) are easily decomposed into C_6_H_5_COOH and C_6_H_5_CHO.

When NH_3_ was added, CIs show a tendency to react with NH_3_ to generate hydroperoxide alkylamine (HPMA and HMA), the saturated vapour pressure of which is not so low as DPSOZ and therefore not so prone to enter into the particle phase as DPSOZ. The rate constants for bimolecular reactions are calculated using conventional transition state theory (TST) (see electronic supplementary material). The results show that the ratio between the reaction rates for C_6_H_5_ĊHOO· reacting with C_6_H_5_CHO and NH_3_ is 10^2^–10^5^. This means that, if the reaction between C_6_H_5_ĊHOO· and NH_3_ plays a leading role compared to that of C_6_H_5_CHO, then the concentration of NH_3_ should be 2 to 5 orders of magnitude larger than the concentration of C_6_H_5_CHO. In the experiments B1–B3, the amount of NH_3_ added in this work was far more than that of aldehydes produced in the reaction. The addition of excess NH_3_ would significantly consume the amount of CI, resulting in a marked decrease in the yield of the final SOA. However, in the experiments B4–B6, even under a smaller [NH_3_]_0_/[styrene]_0_ ratio, the SOA yield was still found to be decreased, indicating that the above competitive reaction mechanism is not the only way of the influence. There may be some other reaction mechanisms which can also reduce the SOA yield after the addition of NH_3_, like the nucleation effect or the organic amine formation. And this needs to be investigated in the further research.

## 4. Conclusion

This work has systematically investigated the effect of NH_3_ on SOA yields from the ozonolysis of styrene using experimental and theoretical methods. Chamber experiments were carried out without NH_3_ or under different [NH_3_]_0_/[styrene]_0_ ratios. The geometry optimization of the stationary points along the reaction pathways were calculated at B3LYP/6-311G++(2d,2p) level, and single point energies have been refined by CBS-QB3 theory. The following conclusions could be drawn from the present work. (i) The addition of NH_3_ could lead to a decrease of SOA yield in the ozonolysis of styrene. And the higher the initial concentration of NH_3_ is, the lower the final SOA yield observed. (ii) The addition of NH_3_ at the beginning of the reaction or several hours after the reaction occurs has obviously different influence on the yield of SOA. (iii) Quantum chemical calculations reveal that a secondary ozonide 3,5-diphenyl-1,2,4-trioxolane (DPSOZ), formed through the reactions of the Criegee intermediate C_6_H_5_ĊHOO· with C_6_H_5_CHO, could make important contribution to the aerosol composition. (iv) The addition of excess NH_3_ would significantly consume the amount of Criegee intermediate, which may decrease the secondary ozonide yield and thus decrease the SOA formation. (v) There may be some other mechanism for the influence of NH_3_ (e.g. the nucleation effect or organic amine formation) on SOA formation, which may be dominant under low-NH_3_ conditions. The findings herein indicate that the reactions of CIs with NH_3_ may play a non-negligible role in locations where the concentration of NH_3_ is relatively high, which could influence the aerosol formation.

## Supplementary Material

Rate constants

## Supplementary Material

Energies

## Supplementary Material

Geometries

## Data accessibility

The datasets supporting this article have been uploaded as part of the electronic supplementary material.
