# Supplementary material for: The influences of ammonia on aerosol formation in the ozonolysis of styrene: roles of Criegee intermediate reactions
Source: R Soc Open Sci. 2018 May 2;5(5):172171. doi: 10.1098/rsos.172171 (PMC5990818; doi:10.1098/rsos.172171)
Supplement: Rate constants [file rsos172171supp1.docx]

**1. The rate constants for bimolecular reactions**

On the basis of the quantum chemical results, the rate constants of the bimolecular reactions between the CI with aldehydes and with NH_3_ were evaluated using conventional transition state theory (TST) which was carried out in the TheRate program [1]. A combined quantum-chemical and the TST approach has been employed to determine the rate constants. The reaction starts to form a pre-reactive complex (RC) and the reaction process is depicted by the equation (I):


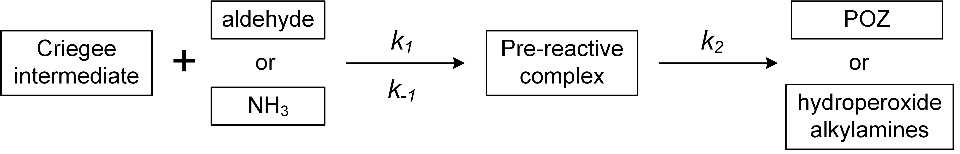
 (I)

It is assumed that the RC is in equilibrium with the reactants and its concentration does not change with time under steady conditions, then the reaction rates are calculated according to equation (II)

$k=\frac{k_{1}}{k_{-1}}k_{2}=K_{eq}k_{2}=\sigma\frac{k_{B}T}{h}\frac{Q^{\neq}}{Q_{R}}e^{-\Delta E^{\neq}/RT}$ (II)

where the *K_eq_* is the equilibrium constant of the pre-reactive complex, *k_2_* is the rate constant of unimolecular reaction between the pre-reactive complex and the product, σ is the symmetry factor, *k_B_* and h are the Bolrzmann and Planck constants, respectively, *Q^≠^* is the partition function of the transition state, *Q_R_* is the partition function of the reactants (R), and ∆*E^≠^* is the barrier height of the reaction.

The rate constants at room temperature (298 K) have been calculated for the reactions of CI with aldehydes and NH_3_. The calculated rate constants at the CBS-QB3 level are depicted in Table S1. The rate constants of CI reaction with aldehydes is on the order of 10^-11^ ~ 10^-10^ (cm^3^ molecule^-1^ s^-1^). For the similar reaction ·CH_2_OO· + CH_3_CHO, an absolute rate coefficient of (9.4 ± 0.7) ⨯ 10^-13^ cm^3^ molecule^-1^ s^-1^ was estimated at 4 Torr in He by direct monitoring of CH_2_OO by Taatjes *et al.* [2]. In the subsequent experiments performed by Stone *et al.* [3], a low pressure limit of ~1.6 ⨯ 10^-29^ cm^3^ molecule^-1^ s^-1^ and a high pressure limit of ~1.7 ⨯ 10^-12^ cm^3^ molecule^-1^ s^-1^ were measured. The theoretical values for CI (C_6_H_5_ĊHOO· or ·CH_2_OO·) + aldehyde (C_6_H_5_CHO or HCHO) on CBS-QB3 level is 1 ~ 3 orders of magnitude larger than the experimental values for ·CH_2_OO· + CH_3_CHO. For ·CH_2_OO· + NH_3_ the rate constant is estimated to be 2.40 ⨯ 10^-14^, which is close to the one under CCSD(T)/6-311+G(d,p) and CCSD(T)/6-311+G(2d,2p) level, and lower than that under B3LYP/6-311++G(2d,2p), CCSD(T)/6-31+G(d) and G3 level calculated by Jørgensen *et al*. [4].

C_6_H_5_CHOO + C_6_H_5_CHO → P_1_ (1)

C_6_H_5_CHOO + NH_3_ → P_2_ (2)

For reaction (1) and (2), the reaction rate is

$$v_{1}=\frac{d[P_{1}]}{dt}=k_{1}\left[ C_{6}H_{5}CHOO \right][C_{6}H_{5}CHO]$$

$$v_{2}=\frac{d[P_{2}]}{dt}=k_{2}\left[ C_{6}H_{5}CHOO \right][NH_{3}]$$

where *v_1_*, *v_2_*, *k_1_* and *k_2_* are the reaction rates and rate constants of reaction (1) and (2). Then the ratio of the rates

$$\frac{v_{1}}{v_{2}}=\frac{k_{1}}{k_{2}}\frac{[C_{6}H_{5}CHO]}{[NH_{3}]}$$

The ratio of rates $\frac{v_{1}}{v_{2}}$ not only depends on the ratio of rate constants $\frac{k_{1}}{k_{2}}$, but also on the ratio of concentrations of reactants $\frac{[C_{6}H_{5}CHO]}{[NH_{3}]}$. The ratio of reaction rate constants $\frac{k_{1}}{k_{2}}$ is approximately four orders of magnitude according to the TST results. Comparing with the previous work discussed above, the ratio $\frac{k_{1}}{k_{2}}$ should be 2 ~ 5 orders of magnitude due to 1 ~ 2 orders of magnitude for theoretical error. Thus, if the reaction between C_6_H_5_ĊHOO· with NH_3_ dominates compared to that with C_6_H_5_CHO, then the ratio $\frac{[C_{6}H_{5}CHO]}{[NH_{3}]}$ should be greater than 10^2^ ~ 10^5^.

Table S1 Calculated rate constants (in units of cm^3^ molecule^-1^ s^-1^) for reactions of CI with aldehydes and NH_3_ at 298 K and atmospheric pressure by CBS-QB3 level of theory.

| reaction | *k^TST^* |
| --- | --- |
| C_6_H_5_ĊHOO· + C_6_H_5_CHO → DPSOZ | 6.64 ⨯ 10^-11^ |
| ·CH_2_OO· + C_6_H_5_CHO → PSOZ | 5.44 ⨯ 10^-11^ |
| C_6_H_5_ĊHOO· + HCHO → PSOZ | 2.42 ⨯ 10^-10^ |
| ·CH_2_OO· + HCHO → HSOZ | 4.03 ⨯ 10^-10^ |
| C_6_H_5_ĊHOO· + NH_3_ → HPMA | 1.65 ⨯ 10^-15^ |
| ·CH_2_OO· + NH_3_ → HMA | 2.40 ⨯ 10^-14^ |

**Reference**

1. Duncan, W. T., Bell, R. L., Truong, T. N. 1998 TheRate: Program for ab initio direct dynamics calculations of thermal and vibrational-state-selected rate constants. J. Comput. Chem., 19: 1039–1052. ([doi:10.1002/(SICI)1096-987X(19980715)19:9<1039::AID-JCC5>3.0.CO;2-R](https://doi.org/10.1002/(SICI)1096-987X(19980715)19:9%3c1039::AID-JCC5%3e3.0.CO;2-R))

2. Taatjes, C. A., Welz, O., Eskola, A. J., Savee, J. D., Osborn, D. L., Lee, E. P. F., Dyke, J. M., Mok, D. W. K., Shallcross, D. E., Percival, C. J. 2012 Direct measurement of Criegee intermediate (CH_2_OO) reactions with acetone, acetaldehyde, and hexafluoroacetone. Phys. Chem. Chem. Phys., 14: 10391-10400. ([doi:10.1039/C2CP40294G](https://doi.org/10.1039/C2CP40294G))

3. Stone, D., Blitz, M., Daubney, L., Howes, N. U. M., Seakins, P. 2014 Kinetics of CH_2_OO reactions with SO_2_, NO_2_, NO, H_2_O and CH_3_CHO as a function of pressure. Phys. Chem. Chem. Phys. 16, 1139-1149. ([doi:10.1039/C3CP54391A](https://doi.org/10.1039/C3CP54391A))

4. Jørgensen, S., Gross, A. 2009 Theoretical investigation of the reaction between carbonyl oxides and ammonia. J. Phys. Chem. A. 113, 10284-10290. ([doi: 10.1021/jp905343u](https://doi.org/10.1021/jp905343u))
