# Supplementary material for: The influences of ammonia on aerosol formation in the ozonolysis of styrene: roles of Criegee intermediate reactions
Source: R Soc Open Sci. 2018 May 2;5(5):172171. doi: 10.1098/rsos.172171 (PMC5990818; doi:10.1098/rsos.172171)
Supplement: Energies [file rsos172171supp2.docx]

**2. Total energies calculated at the CBS-QB3 level.**

Table S2. Total energies calculated at the CBS-QB3 level.

| species | CBS-QB3 (0 K) | species | CBS-QB3 (0 K) |
| --- | --- | --- | --- |
| C6H5CHO | -344.974798 | TS_DPC | -764.96238 |
| C6H5CHOO | -419.975629 | TS_DPD1 | -764.957704 |
| HCHO | -114.344172 | TS_DPD2 | -764.961253 |
| CH2OO | -189.343124 | TS_DPI1 | -764.957191 |
| [C6H5CHOO+C6H5CHO] | -764.964921 | TS_DPI2 | -765.132172 |
| [CH2OO+C6H5CHO] | -534.330418 | TS_DPI3 | -765.117057 |
| [C6H5CHOO+HCHO] | -534.332598 | TS_PC1 | -534.328652 |
| [CH2OO+HCHO] | -303.695214 | TS_PC2 | -534.33134 |
| DPSOZ | -765.019194 | TS_PD1 | -534.328801 |
| PSOZ | -534.393816 | TS_PD2 | -534.330759 |
| HSOZ | -303.76927 | TS_PI1 | -534.337206 |
| HPMB1 | -765.134834 | TS_PI2 | -534.511742 |
| HPMB2 | -765.141564 | TS_PI3 | -534.488476 |
| HMB1 | -534.515935 | TS_C | -303.694957 |
| HMB2 | -534.521003 | TS_D | -303.70199 |
| HMF1 | -303.88677 | TS_I1 | -303.713192 |
| HMF2 | -303.8909 | TS_I2 | -303.88337 |
| C6H5COOH | -420.155851 | TS_I3 | -303.856458 |
| HCOOC6H5 | -420.126583 | TS_NC1 | -476.435012 |
| HCOOH | -189.520985 | TS_NC2 | -245.803889 |
| NH3 | -56.460189 | TSND1 | -476.420191 |
| [C6H5CHOO+NH3] | -476.443982 | TS_ND2 | -245.788475 |
| [CH2OO+NH3] | -245.810401 |  |  |
| HPMA | -476.494962 |  |  |
| HMA | -245.873394 |  |  |
| C6H5CH=NH | -325.090509 |  |  |
| CH2=NH | -94.465261 |  |  |
