# Supplementary material for: The influences of ammonia on aerosol formation in the ozonolysis of styrene: roles of Criegee intermediate reactions
Source: R Soc Open Sci. 2018 May 2;5(5):172171. doi: 10.1098/rsos.172171 (PMC5990818; doi:10.1098/rsos.172171)
Supplement: Geometries [file rsos172171supp3.docx]

**3. Geometries optimized at the B3LYP/6-311G++(2d,2p).**

C6H5CHO

0 1

O 2.84395000 -0.39219800 0.00004200

C 1.98864200 0.46366800 -0.00004500

C 0.53271700 0.20555400 -0.00002000

C -0.35413600 1.28395900 -0.00000100

C -1.72613100 1.06196100 0.00001000

C -2.21264300 -0.24155700 0.00001300

C -1.33048900 -1.32326100 -0.00000200

C 0.03756400 -1.10280400 -0.00001700

H 2.26767000 1.53598700 0.00002000

H 0.03565900 2.29438600 -0.00000200

H -2.41215400 1.89749700 0.00002000

H -3.27973100 -0.41775400 0.00002300

H -1.71631400 -2.33335500 -0.00000200

H 0.74011400 -1.92430600 -0.00003000

C6H5CHOO

0 1

O 3.61997800 -0.03014600 0.00000000

O 2.29828500 -0.39002000 -0.00000100

C 1.43254100 0.53219400 0.00000000

C 0.02838500 0.22387400 0.00000000

C -0.88434100 1.29072500 0.00000000

C -2.24930100 1.04384200 0.00000000

C -2.71791300 -0.26633100 0.00000000

C -1.81703300 -1.33311300 0.00000000

C -0.45402300 -1.09757700 0.00000000

H 1.80757800 1.55055800 0.00000100

H -0.51621600 2.30804100 0.00000000

H -2.94613600 1.87005000 -0.00000100

H -3.78170800 -0.45921300 -0.00000100

H -2.18523200 -2.34943500 0.00000000

H 0.24572100 -1.92036500 0.00000100

HCHO

0 1

C -0.52757700 -0.00000300 -0.00000900

O 0.67328600 -0.00019500 0.00000200

H -1.10892800 0.93945500 0.00001800

H -1.11190000 -0.93787700 0.00001800

CH2OO

0 1

O -1.17750300 -0.20304800 0.00000000

O 0.00000000 0.45957200 0.00000000

C 1.06986500 -0.19534700 0.00000000

H 1.97309900 0.39729400 0.00000000

H 1.02773600 -1.27739600 0.00000000

[C6H5CHOO+C6H5CHO]

0 1

O -1.17765900 -1.48843500 0.05433100

C -2.09023400 -0.71232100 -0.16496600

C -2.51048400 0.36012900 0.76243400

C -3.55525100 1.21003900 0.39693500

C -3.97471800 2.21522200 1.25941200

C -3.34978400 2.37148800 2.49305700

C -2.30537900 1.52428300 2.86413700

C -1.88581300 0.52210900 2.00253900

H -2.69345300 -0.79977800 -1.08138300

H -4.02592200 1.08680800 -0.56984200

H -4.78081800 2.87598600 0.97205000

H -3.67480400 3.15282200 3.16683700

H -1.82476100 1.64953100 3.82484700

H -1.08099500 -0.14768700 2.27102200

O -0.83913400 0.71197300 -2.15841700

O 0.43067000 0.57414900 -1.63420600

C 0.91321900 -0.59293300 -1.58900100

C 2.23125700 -0.81669800 -1.05513800

C 2.70690300 -2.13553000 -1.01086400

C 3.97150100 -2.40523900 -0.50874400

C 4.77139500 -1.36405300 -0.04805900

C 4.30433800 -0.04889900 -0.08755000

C 3.04408200 0.22962600 -0.58561400

H 0.30018300 -1.39790500 -1.97338200

H 2.07623900 -2.94066300 -1.36235100

H 4.33204600 -3.42355200 -0.47452800

H 5.75750400 -1.57356200 0.34284300

H 4.92959200 0.75630700 0.27191200

H 2.67809600 1.24532200 -0.61882200

[CH2OO+C6H5CHO]

0 1

O 0.89305600 -1.63107200 -0.90997700

C -0.10931900 -0.93700000 -0.87740200

C -0.72727300 -0.42111500 0.36078100

C -1.84047100 0.41618600 0.27007500

C -2.44078400 0.90930700 1.42183600

C -1.93171000 0.56045700 2.66913100

C -0.82230800 -0.27995500 2.76598100

C -0.22064600 -0.76895200 1.61674100

H -0.64399800 -0.68456000 -1.80616500

H -2.22212100 0.68822300 -0.70552200

H -3.29861500 1.56330300 1.34955700

H -2.39815800 0.94187400 3.56738500

H -0.43384600 -0.55040300 3.73827600

H 0.63675100 -1.42510100 1.66997900

O 1.11954100 1.35251400 -1.80031900

O 2.03467400 0.61642300 -2.50332000

C 2.84758500 -0.07581800 -1.85264900

H 3.52513900 -0.67163300 -2.44788100

H 2.84477600 -0.03749500 -0.77296500

[C6H5CHOO+HCHO]

0 1

O 0.85000800 -3.34894700 -0.22559300

C 0.22809600 -0.76986300 -0.30021300

C -0.39652900 -0.25172900 0.88828900

C -1.76021000 -0.51593900 1.08137100

C -2.40933900 -0.03920100 2.21053600

C -1.70445400 0.70300600 3.15310800

C -0.34622000 0.96826000 2.96837200

C 0.30990500 0.49524500 1.84627300

H -0.32247000 -1.33395000 -1.04101100

H -2.29879500 -1.10116600 0.34857300

H -3.45954800 -0.24746400 2.35741800

H -2.20967400 1.07404000 4.03412000

H 0.19623300 1.54313100 3.70559900

H 1.36143200 0.69346500 1.69965400

O 1.44961800 -0.53722800 -0.53381400

O 1.97625600 -1.11101100 -1.67983900

C 1.80949800 -3.36060200 -0.97395400

H 1.70862800 -3.58390600 -2.04545700

H 2.83692000 -3.25605400 -0.59809700

[CH2OO+HCHO]

0 1

O -1.07759700 -1.19470200 -0.00098500

O -1.83149400 -0.06037600 0.00105300

C -1.25713100 1.05321600 -0.00027000

H -1.92841400 1.90131400 0.00169700

H -0.17049600 1.10748300 -0.00277700

C 2.13318200 -0.48313300 0.00066100

O 1.96005500 0.71225700 -0.00054400

H 3.15311800 -0.90548100 0.00242000

H 1.28178100 -1.18124500 0.00012000

DPSOZ

0 1

O -1.85990000 -4.22606900 -0.98696000

C -2.94951400 -3.40751000 -1.39653800

C -3.20436400 -2.27590100 -0.42020900

C -3.66426400 -1.04286100 -0.87746600

C -3.95198400 -0.02471000 0.02593200

C -3.79263300 -0.23490300 1.39166900

C -3.34005300 -1.46796600 1.85145600

C -3.04629300 -2.48500000 0.94952000

H -3.84070500 -4.02622000 -1.53037800

H -3.77916400 -0.87749400 -1.93905400

H -4.29767200 0.93306500 -0.33847700

H -4.01736800 0.55700900 2.09300600

H -3.21187900 -1.63756400 2.91193400

H -2.68209100 -3.43836400 1.30532700

O -2.61181600 -2.97486400 -2.71099000

O -1.16828200 -2.81134400 -2.58890900

C -0.83225600 -4.04262100 -1.96148400

C 0.53798100 -3.93518000 -1.35896100

C 1.56583900 -4.73799900 -1.84572500

C 2.84604000 -4.64134800 -1.30859300

C 3.09959900 -3.73762500 -0.28355100

C 2.07288200 -2.93108800 0.20398400

C 0.79540000 -3.02840200 -0.33019000

H -0.89007200 -4.85216600 -2.69392700

H 1.36512200 -5.44053800 -2.64404900

H 3.63953900 -5.26955800 -1.68891500

H 4.09323100 -3.66069000 0.13645600

H 2.27014200 -2.22763200 1.00115500

H -0.00505200 -2.40880600 0.04714000

PSOZ

0 1

O 0.88333400 -1.69480100 -0.53867400

C -0.04677600 -0.67782700 -0.90990900

C -0.59199300 0.08619600 0.26122100

C -1.95395800 0.36890300 0.31933100

C -2.47446600 1.09987300 1.38275200

C -1.63136500 1.54811900 2.39287800

C -0.26809800 1.26472100 2.33879100

C 0.25100000 0.53652300 1.27711100

H -0.84478700 -1.12731400 -1.50634600

H -2.60847800 0.01637900 -0.46709500

H -3.53325300 1.31505600 1.42244900

H -2.03333600 2.11400300 3.22215500

H 0.38766500 1.61038300 3.12601200

H 1.30593200 0.30752600 1.23556400

O 0.71461400 0.20659400 -1.73037400

O 1.48876000 -0.75906500 -2.50227800

C 1.95873000 -1.58876800 -1.45788500

H 2.19241800 -2.55606200 -1.90185600

H 2.82516600 -1.14573400 -0.96037300

HSOZ

0 1

O -0.66860300 -0.94909900 0.29200600

O 0.67049400 -0.94766000 -0.29304000

C 1.11842800 0.32197900 0.13563800

H 1.92086600 0.61917500 -0.53801700

H 1.44044900 0.29583500 1.17936200

C -1.11942000 0.32066700 -0.13403700

O -0.00067300 1.18647700 -0.00113700

H -1.44521700 0.29569700 -1.17657500

H -1.91989500 0.61566700 0.54299800

HPMB1

0 1

O -0.31304900 -0.59842700 -1.04333000

C -1.56994200 0.10357800 -1.27317400

C -2.15715900 0.70690000 -0.01662400

C -2.51675200 2.04919600 0.05368800

C -3.09679800 2.55479800 1.21234800

C -3.32307700 1.72538500 2.30556000

C -2.96920600 0.38139500 2.23562500

C -2.39368900 -0.12570400 1.07699000

H -2.20155500 -0.72252800 -1.60550400

H -2.33078800 2.69357500 -0.79131300

H -3.36806300 3.60057400 1.26130000

H -3.77308200 2.12215600 3.20535500

H -3.14358600 -0.27157000 3.07984800

H -2.12004900 -1.17165600 1.02619500

O -1.44388700 1.06067400 -2.27523000

O 0.72187800 1.24665200 -0.22403200

C 0.74663100 0.08073600 -0.52002300

C 1.94228900 -0.79918900 -0.35900100

C 1.93631900 -2.15233500 -0.70652100

C 3.08018200 -2.92127800 -0.52914400

C 4.23430100 -2.34592100 -0.00577900

C 4.24403600 -0.99732100 0.34176300

C 3.10337800 -0.22657900 0.16605800

H -1.24641400 0.61607300 -3.10458100

H 1.03957000 -2.59549400 -1.11134300

H 3.07121200 -3.96835000 -0.79899700

H 5.12336500 -2.94640300 0.13132400

H 5.13968500 -0.54897100 0.74907400

H 3.09085400 0.82063100 0.43055600

HPMB2

0 1

O -0.79700300 -0.41158000 -0.83781900

C -1.67890000 0.75270200 -0.72630700

C -1.62805400 1.33442700 0.67878000

C -1.85200200 2.69599100 0.87368700

C -1.87658500 3.22825900 2.15905600

C -1.68525700 2.40401100 3.26199600

C -1.47271900 1.04183600 3.07315700

C -1.44896900 0.50901200 1.78958200

H -2.65700600 0.31358900 -0.91205100

H -2.00772600 3.33715600 0.01839400

H -2.04487800 4.28771400 2.29623100

H -1.70202600 2.81825700 4.26072500

H -1.32374600 0.39286900 3.92537500

H -1.28265500 -0.54992000 1.65154700

O -1.46614800 1.66393700 -1.74632300

O 1.01494300 0.93214000 -0.88268100

C 0.52850300 -0.18069600 -0.89802200

C 1.32946000 -1.43132700 -0.99643300

C 0.73473200 -2.69444400 -1.05477200

C 1.52801800 -3.83095300 -1.14802400

C 2.91457400 -3.71370700 -1.18156100

C 3.51022200 -2.45612700 -1.12308000

C 2.72141100 -1.31806200 -1.03198000

H -0.57118900 2.01492700 -1.62720100

H -0.34083800 -2.77984600 -1.03244400

H 1.06545300 -4.80700800 -1.19600900

H 3.52985700 -4.60021700 -1.25411500

H 4.58716400 -2.36461600 -1.14942600

H 3.16454100 -0.33412500 -0.98695000

HMB1

0 1

O 1.34644500 -0.52064800 -0.94212400

C 0.17485700 0.17709600 -0.92641100

C -0.36574400 0.34161200 0.45113700

C -1.55837100 1.05321600 0.60346100

C -2.10509400 1.23520700 1.86583900

C -1.46501800 0.70785400 2.98475000

C -0.27690800 -0.00201300 2.83795900

C 0.27355100 -0.18647500 1.57568600

H 1.79079000 -2.74868400 -2.17958000

H -2.04023600 1.45354400 -0.27641800

H -3.02837300 1.78629000 1.97885400

H -1.89141500 0.84972400 3.96854800

H 0.22039700 -0.41132400 3.70642100

H 1.19496000 -0.73540600 1.45751300

O -0.34700100 0.61899000 -1.91909800

O 1.44863500 -2.03464400 -2.72601300

C 1.90993800 -0.81688800 -2.23196500

H 2.98550300 -0.81862800 -2.06040700

H 1.61628000 -0.05247700 -2.94216200

HMB2

0 1

O 0.91944800 0.65815200 -1.33888900

C -0.26975500 0.32811200 -0.79494400

C -0.16884500 -0.09187800 0.62701000

C -1.34255700 -0.45711200 1.29109200

C -1.29488300 -0.85354800 2.62004200

C -0.07599000 -0.88893500 3.29297700

C 1.09573500 -0.52676900 2.63454500

C 1.05262900 -0.12822700 1.30466500

H -0.44697000 -0.05587300 -3.46763600

H -2.27792400 -0.42419500 0.75195400

H -2.20478300 -1.13540600 3.13140100

H -0.03962800 -1.19909300 4.32846100

H 2.04188600 -0.55539400 3.15686500

H 1.95762100 0.15255300 0.78827900

O -1.30946000 0.37233900 -1.42141700

O 0.49329700 0.11861700 -3.59846500

C 0.85180400 1.11963200 -2.71276000

H 1.86625600 1.42539300 -2.94255300

H 0.16429100 1.96497300 -2.73986500

HMF1

0 1

O 1.71067200 -0.52918200 -0.40219300

O -1.60148700 -0.82035100 0.10401900

C -1.30561300 0.28980200 -0.23148700

H -1.97875200 0.98950000 -0.74020200

H 2.21934300 0.12835900 -0.88657400

C 0.93784800 0.10403200 0.56294900

O -0.11380900 0.89523900 -0.03896100

H 0.49780500 -0.66885100 1.18285300

H 1.50518200 0.82234200 1.15223500

HMF2

0 1

O -1.49543000 0.65988800 -0.36100700

O 1.31204700 0.89914300 0.23327700

C 1.26017200 -0.23615700 -0.16720500

H 2.11070200 -0.78367900 -0.58766600

H -0.90451300 1.41112400 -0.22961900

C -1.02140000 -0.39034700 0.40123400

O 0.17010100 -1.00832700 -0.17155600

H -0.77105800 -0.09230100 1.41915700

H -1.76150100 -1.18174700 0.38823900

C6H5COOH

0 1

O -1.32988800 -1.59498700 -0.46624500

C -1.83017000 -0.51730800 -0.68446700

C -2.13017600 0.50724200 0.34798600

C -2.71025900 1.73721300 0.02688600

C -2.97207100 2.66208800 1.02987800

C -2.65740200 2.36482600 2.35274100

C -2.07899700 1.13940000 2.67528800

C -1.81550500 0.21263300 1.67690600

H -1.94048100 -0.86367000 -2.52444300

H -2.95268800 1.96251400 -1.00037300

H -3.42095500 3.61342100 0.78027400

H -2.86232700 3.08683600 3.13149700

H -1.83451600 0.90899000 3.70286800

H -1.36742100 -0.74318700 1.90572500

O -2.17690600 -0.13066000 -1.93945100

HCOOC6H5

0 1

O 2.61103000 0.86059300 0.53210300

O 1.41923600 -0.87624400 -0.38112400

C 2.53841000 -0.21711100 0.00375500

C 0.13231100 -0.35879400 -0.17912800

C -0.18570700 0.99418800 -0.30018500

C -1.51836300 1.38131200 -0.14332600

C -2.51244200 0.43903500 0.12404100

C -2.17357400 -0.91111800 0.23477200

C -0.84814400 -1.31490400 0.08255900

H 3.39845600 -0.85236100 -0.25465200

H 0.58480700 1.72641400 -0.49908500

H -1.77558700 2.43315500 -0.23231100

H -3.54485500 0.75458400 0.24412600

H -2.93970900 -1.65374700 0.43917800

H -0.56017200 -2.35848600 0.15999700

HCOOH

0 1

O 1.05711600 -0.27890300 -0.00001200

C -0.13405900 0.36168000 0.00001000

H 1.77647700 0.36340900 0.00006800

H -0.04340000 1.45986900 -0.00004800

O -1.17320600 -0.22026700 0.00000200

TS_DPC

0 1

O -1.69307500 -3.81208400 -0.59315700

C -2.76758900 -3.24476900 -0.91810900

C -3.42270600 -2.25839700 -0.02495600

C -4.71830600 -1.82566100 -0.30970300

C -5.35224100 -0.91078700 0.52190800

C -4.69334400 -0.42472400 1.64748100

C -3.40184400 -0.85924800 1.93994700

C -2.76914200 -1.77429500 1.10972000

H -3.42636200 -3.70336100 -1.66346600

H -5.22448300 -2.20195700 -1.18960400

H -6.35514300 -0.57663500 0.29387200

H -5.18491300 0.28726900 2.29656400

H -2.89294400 -0.48550100 2.81817700

H -1.77229800 -2.12920200 1.32831300

O -2.10649700 -2.00777700 -2.42182600

O -0.74266000 -2.11791200 -2.14134000

C -0.37168600 -3.34827400 -2.12786500

C 0.96786100 -3.67000800 -1.67719300

C 1.47848700 -4.94130000 -1.96059200

C 2.75927100 -5.28514100 -1.55165600

C 3.53538900 -4.36167600 -0.85789800

C 3.02908800 -3.09490000 -0.56785500

C 1.74929200 -2.74748400 -0.96841100

H -0.93504400 -4.04759900 -2.72745200

H 0.86756700 -5.65539200 -2.49609300

H 3.15072700 -6.26832600 -1.77121900

H 4.53316900 -4.62866800 -0.53779000

H 3.63382700 -2.38331400 -0.02343700

H 1.34502200 -1.77296400 -0.73781600

TS_DPD1

0 1

O -2.04378300 -4.54493700 -0.86196100

C -2.86808000 -3.58059200 -1.14083200

C -3.20486200 -2.57435100 -0.05048900

C -3.87272600 -1.38966300 -0.35898000

C -4.18288400 -0.48416400 0.64846900

C -3.84243100 -0.76569200 1.96911900

C -3.18624700 -1.95344600 2.27858100

C -2.86934900 -2.85869800 1.27164600

H -3.90271600 -3.95763400 -1.56430300

H -4.13161000 -1.17571000 -1.38634600

H -4.69011000 0.43924100 0.40444700

H -4.08863400 -0.06235700 2.75299900

H -2.91914100 -2.17391800 3.30314700

H -2.35339900 -3.78112000 1.49357400

O -2.79935700 -3.11452500 -2.41908600

O -0.98140900 -2.69946700 -2.34074400

C -0.53543400 -3.84636200 -2.03524200

C 0.63846800 -3.96112300 -1.14504100

C 1.29639200 -5.18879900 -1.05884500

C 2.41591600 -5.32359100 -0.24751600

C 2.88152300 -4.23118400 0.47780600

C 2.22740800 -3.00315800 0.39072300

C 1.10974100 -2.86453900 -0.41867000

H -0.74418700 -4.69272600 -2.68639900

H 0.92435900 -6.03537800 -1.62039800

H 2.92231600 -6.27637000 -0.18010900

H 3.75359500 -4.33458700 1.10897500

H 2.59248500 -2.15544000 0.95386500

H 0.59463600 -1.91833900 -0.49626900

TS_DPD2

0 1

O -1.91660500 -4.43924600 -0.75621200

C -3.18556600 -3.46683700 -1.46628900

C -3.29381500 -2.30677600 -0.51897300

C -2.88330600 -1.03527100 -0.91419800

C -3.00800800 0.04026600 -0.04166500

C -3.54689700 -0.14697000 1.22842100

C -3.96577300 -1.41493500 1.62371100

C -3.84176200 -2.48919100 0.75076600

H -3.95087700 -4.23712900 -1.30864600

H -2.47532500 -0.90468100 -1.90597400

H -2.69020300 1.02601100 -0.35386600

H -3.64677700 0.69146200 1.90448900

H -4.39122200 -1.56374000 2.60693900

H -4.16548200 -3.47596400 1.05679900

O -2.86553200 -3.28368400 -2.69722200

O -0.87308900 -3.25667100 -2.35533600

C -0.98815300 -4.41756700 -1.69007300

C 0.50245100 -3.91700400 -1.29118500

C 1.58851300 -4.37114600 -2.04171000

C 2.87239100 -4.13913600 -1.57190300

C 3.06153200 -3.43138300 -0.38608800

C 1.96660700 -2.95145200 0.33561900

C 0.67892100 -3.17650800 -0.12018600

H -0.89877900 -5.32491400 -2.30010200

H 1.42058000 -4.90118700 -2.96812400

H 3.72363500 -4.50050900 -2.13130300

H 4.06409300 -3.24703500 -0.02563600

H 2.12192000 -2.40087500 1.25269100

H -0.18648600 -2.81925600 0.41682000

TS_DPI1

0 1

O -1.68952500 -3.99339700 -1.07861500

C -2.86545500 -3.30618600 -1.73188900

C -3.74809000 -2.68548900 -0.64659400

C -3.64188600 -1.32716300 -0.36044900

C -4.44268300 -0.76960200 0.62914600

C -5.35153900 -1.56012500 1.32776400

C -5.45920100 -2.91555500 1.03506500

C -4.65939800 -3.47732900 0.04510000

H -3.39565500 -4.19008100 -2.13734600

H -2.93196900 -0.72495000 -0.90726500

H -4.35787300 0.28486400 0.85425900

H -5.97469400 -1.12062100 2.09459200

H -6.16554700 -3.53407600 1.57167200

H -4.74422500 -4.53194100 -0.18349500

O -2.48114300 -2.53189200 -2.70007900

O -0.53282400 -2.07644400 -1.48243700

C -0.55687800 -3.36067600 -1.57365500

C 0.73340100 -4.15258600 -1.38417000

C 0.70158000 -5.54321700 -1.48601900

C 1.86962700 -6.27128800 -1.29824400

C 3.06930600 -5.61314000 -1.04199300

C 3.09766300 -4.22271200 -0.96413700

C 1.93292500 -3.48640800 -1.14001900

H -0.71562900 -3.52026600 -2.72163500

H -0.23254000 -6.04540400 -1.69071900

H 1.84398100 -7.35030200 -1.36078900

H 3.97874500 -6.18125600 -0.90340700

H 4.02669200 -3.71066000 -0.75480700

H 1.93842400 -2.40929900 -1.07140500

TS_DPI2

0 1

O -0.65781700 -0.94240500 -1.58680400

C -1.61408600 0.12142200 -1.32834600

C -1.49049500 0.69069200 0.07034300

C -1.07578000 1.99893500 0.29901000

C -0.99589800 2.48650800 1.59923100

C -1.32638000 1.67150900 2.67695800

C -1.74578500 0.36360100 2.45153500

C -1.83357300 -0.12080100 1.15207000

H -2.55568900 -0.42408100 -1.41009400

H -0.81460700 2.62220600 -0.54175700

H -0.67142500 3.50392500 1.77013200

H -1.26221800 2.05364800 3.68669600

H -2.01034400 -0.27393700 3.28401900

H -2.16875500 -1.13570400 0.97813400

O -1.57840100 1.12063900 -2.30997100

O 1.14656100 0.42135200 -1.45376800

C 0.67932000 -0.68688100 -1.53802300

C 1.48856800 -1.93477600 -1.63351600

C 0.90841500 -3.20266200 -1.72543400

C 1.71501700 -4.33062700 -1.81111100

C 3.10066300 -4.20050800 -1.80415700

C 3.68220200 -2.93833600 -1.71159300

C 2.88016100 -1.80913800 -1.62743600

H -0.96856300 0.86528500 -3.00594100

H -0.16624800 -3.29932900 -1.73003500

H 1.26318100 -5.31028800 -1.88268900

H 3.72619700 -5.08032800 -1.87031900

H 4.75853500 -2.83646900 -1.70522200

H 3.31226400 -0.82191000 -1.55415300

TS_DPI3

0 1

O -0.59035400 -1.01781300 -1.60845000

C -2.01454100 0.19985900 -1.46825800

C -2.14494600 0.24236800 0.01151500

C -1.69354100 1.34400500 0.73953500

C -1.84140500 1.37183300 2.12046700

C -2.44509500 0.30476000 2.78026400

C -2.90710500 -0.79194900 2.05660200

C -2.75976300 -0.82069600 0.67672600

H -2.71639900 -0.47046400 -1.96486000

H -1.24253700 2.17552600 0.21790200

H -1.49256000 2.22749900 2.68193200

H -2.56233200 0.33064500 3.85506500

H -3.38279300 -1.61763500 2.56745500

H -3.11591700 -1.67109300 0.10981900

O -1.62671000 1.22706600 -2.14176500

O 0.68501000 0.80657800 -1.86113500

C 0.53951700 -0.45563500 -1.77645000

C 1.74569600 -1.32224600 -1.87741400

C 1.63525600 -2.70706800 -1.73402000

C 2.76596900 -3.50704100 -1.82995400

C 4.01031600 -2.92909900 -2.06834200

C 4.12342800 -1.54859300 -2.21091800

C 2.99488400 -0.74510900 -2.11581300

H -0.47441100 1.24774200 -1.98798200

H 0.66428200 -3.14145300 -1.54864000

H 2.67894100 -4.57904400 -1.71906400

H 4.89025700 -3.55358800 -2.14313800

H 5.08969400 -1.10044700 -2.39673000

H 3.06554800 0.32681100 -2.22473300

TS_PC1

0 1

O 1.18480000 -1.47288300 -0.59917200

C 0.15877900 -0.76468200 -0.51872500

C -0.40293900 -0.29613600 0.76527900

C -1.54573100 0.50686200 0.76606700

C -2.09260500 0.94635300 1.96397600

C -1.50528800 0.57777500 3.17179100

C -0.37235700 -0.23299300 3.17838600

C 0.17643100 -0.67022900 1.98036800

H -0.49268200 -0.66496600 -1.39214700

H -1.99244300 0.79592700 -0.17637000

H -2.97305200 1.57394500 1.95896900

H -1.93256600 0.91663500 4.10574400

H 0.07491400 -0.52853400 4.11762900

H 1.04112500 -1.31914200 1.97129400

O 1.02764100 1.17489200 -1.16595300

O 2.17733000 0.58590700 -1.67022400

C 2.77507500 -0.11163100 -0.80088500

H 3.59233900 -0.71105100 -1.17805300

H 2.63108600 0.09619500 0.24756800

TS_PC2

0 1

O 0.94957100 -2.94662800 0.04151400

C 0.22001200 -0.86834800 -0.33304800

C -0.39807100 -0.33156800 0.85528300

C -1.77239300 -0.53349600 1.03486200

C -2.40780400 -0.03339700 2.16188300

C -1.67549700 0.66852800 3.11470300

C -0.30497800 0.86747300 2.94431100

C 0.33701200 0.36743300 1.82457400

H -0.35525000 -1.39539900 -1.07847800

H -2.33215000 -1.08717000 0.29339700

H -3.46787400 -0.19214300 2.29957200

H -2.16968200 1.05664500 3.99470900

H 0.25938300 1.40650600 3.69206200

H 1.40006400 0.50356800 1.69231100

O 1.41279300 -0.51884800 -0.63303000

O 1.93872800 -1.26083600 -1.68803300

C 1.79642900 -3.07594200 -0.86434200

H 1.52522200 -3.50236300 -1.83798700

H 2.86384100 -3.10392900 -0.61892700

TS_PD1

0 1

O 0.57321800 -1.99869500 -0.95932600

C 0.41827600 0.11883200 -0.45670100

C -0.24633200 0.11689700 0.85307600

C -1.63877100 0.01234700 0.89347600

C -2.30219600 0.02961500 2.11284600

C -1.57659400 0.15255000 3.29409700

C -0.18670600 0.25919200 3.25694200

C 0.47986300 0.24336400 2.04179600

H -0.21202600 0.12629400 -1.34278800

H -2.19329500 -0.09376200 -0.02917000

H -3.37931100 -0.05591800 2.14329100

H -2.09249600 0.16565900 4.24445000

H 0.37240400 0.35575800 4.17714900

H 1.55587500 0.32758900 1.99857000

O 1.63863700 0.41238000 -0.55769100

O 2.15151800 -0.69581700 -1.94934900

C 1.80092100 -1.85961100 -1.33066300

H 2.06685500 -2.33835300 -2.35557900

H 2.57857000 -2.23824300 -0.64083900

TS_PD2

0 1

O 1.09195600 -1.71801900 -0.39565700

C 0.08925500 -0.96369200 -0.80431700

C -0.57253800 0.12923900 0.21163600

C -1.89933400 0.52157200 0.00683000

C -2.55758300 1.21371000 1.01030400

C -1.88731500 1.53361700 2.19054500

C -0.55279300 1.16280600 2.37170100

C 0.11867500 0.47330000 1.37818900

H -0.72201800 -1.48747800 -1.32174000

H -2.39372300 0.27867300 -0.92269700

H -3.58735100 1.51142900 0.87261400

H -2.40259500 2.07882200 2.96904900

H -0.04069700 1.41599900 3.28920100

H 1.14853300 0.17154200 1.49329300

O 0.42701600 0.24281800 -1.27369200

O 1.88343800 -0.71222300 -2.44749600

C 2.34355100 -1.30765800 -1.40798400

H 2.71127600 -2.32775500 -1.58046300

H 2.97452600 -0.74284900 -0.70976600

TS_PI1

0 1

O 2.65977600 -1.00775200 0.00137400

C 1.25283800 -1.01414500 0.01258500

C 0.65589400 -0.66143700 1.36400500

C -0.52182000 0.08114100 1.43493000

C -1.05399900 0.39999300 2.67742800

C -0.42166000 -0.02671800 3.84330900

C 0.74810000 -0.77754800 3.76651400

C 1.28082900 -1.11087500 2.52772500

H 1.08379800 -2.17443500 -0.02074100

H -0.99496700 0.41392900 0.52344200

H -1.95711100 0.99154900 2.73674500

H -0.83992200 0.22495600 4.80810300

H 1.23945800 -1.11233800 4.66941800

H 2.18341500 -1.70065800 2.45912500

O 0.68155800 -0.62552400 -1.06917400

O 2.10237100 -2.39799700 -1.76074200

C 3.06379400 -1.73086900 -1.19977100

H 3.88603600 -2.38259100 -0.84572200

H 3.53309100 -0.98010100 -1.86909400

TS_PI2

0 1

O 1.95475900 -0.84110700 -0.79877000

C 0.86315900 -0.39919900 -0.10164100

C 1.07260700 -0.47576700 1.36895800

C 0.02399900 -0.06930000 2.19847700

C 0.16746700 -0.12054600 3.57755500

C 1.35822700 -0.57730300 4.13732800

C 2.40543300 -0.98268100 3.31500100

C 2.26618700 -0.93374600 1.93366500

H 1.36387800 -2.68126700 -1.99176400

H -0.89120600 0.28280700 1.74563000

H -0.64598200 0.19472400 4.21604400

H 1.46971900 -0.61661800 5.21237500

H 3.32998700 -1.33645700 3.74963500

H 3.07604500 -1.24550500 1.29245100

O -0.14164600 0.00344200 -0.63004400

O 1.54861100 -2.09474200 -2.72993700

C 1.87888600 -0.82301400 -2.22594000

H 2.87722700 -0.55247600 -2.56503300

H 1.13665400 -0.09438500 -2.54196500

TS_PI3

0 1

O 1.95635500 -1.29860300 -0.79327600

C 0.83776100 -1.62831000 -0.29079500

C 0.78423400 -2.01244800 1.14078200

C -0.42458800 -2.42943100 1.70378900

C -0.47605300 -2.78846300 3.04373300

C 0.67533200 -2.73302400 3.82483000

C 1.88132800 -2.31727100 3.26575800

C 1.93797500 -1.95676200 1.92673700

H 0.06729600 -1.56199000 -2.08316400

H -1.30848600 -2.46855000 1.08507300

H -1.41123100 -3.11198300 3.47902400

H 0.63305300 -3.01384700 4.86842200

H 2.77429300 -2.27476900 3.87361900

H 2.86589700 -1.63314900 1.47944500

O -0.25261200 -1.64049800 -0.96490100

O 0.86821700 -1.54171900 -3.04789400

C 1.79866000 -0.82867800 -2.54910600

H 2.82745600 -1.04271000 -2.84004600

H 1.60484200 0.21669000 -2.29023900

TS_C

0 1

O 0.08569700 1.50629000 -0.51646000

O 1.30439900 1.56706300 0.08911000

C 2.05717000 0.56741800 0.03755000

H 3.00637800 0.69062700 0.54055100

H 1.72292400 -0.33671300 -0.45637200

C -1.03769400 -1.30896800 0.07556300

O 0.04764800 -1.78397600 0.30446600

H -1.89991000 -1.51499600 0.73392600

H -1.21188600 -0.64821400 -0.78485100

TS_D

0 1

O -1.03798000 -0.98532200 0.40522000

O 0.81144100 -1.00478200 -0.39183400

C 1.04838700 0.19167000 0.15051900

H 1.92231800 0.01436800 -0.65155400

H 1.54661900 0.20194600 1.13246200

C -1.34861800 0.12139700 -0.10753600

O 0.21375100 1.14350400 -0.11568700

H -1.52047700 0.21641300 -1.18111200

H -1.86335200 0.84235800 0.53579900

TS_I1

0 1

O -0.71881100 -0.71819000 1.08487500

O 1.14112500 -0.78822500 -0.46246500

C 1.13607500 0.36452200 0.08723700

H 2.06183900 0.95723500 0.05446200

H 1.00458900 0.08611100 1.23762800

C -1.11300400 0.35408300 0.46466200

O -0.00496600 1.15510600 -0.05222500

H -1.80295300 0.13940500 -0.37644800

H -1.63934100 1.07202000 1.12169100

TS_I2

0 1

O 1.78752200 -0.71962000 -0.29301100

O -1.43948100 0.67640700 -0.82295900

C -0.54790400 1.47282900 -0.80002200

H -0.57842100 2.46328400 -1.26758000

H 1.92437100 -0.30123400 -1.14710400

C 0.92086800 0.07659000 0.47230700

O 0.65640200 1.32033400 -0.19886200

H -0.01687400 -0.44458200 0.64831600

H 1.41116500 0.34737100 1.40510600

TS_I3

0 1

O -1.24051400 0.78347100 -0.15839900

O 1.11947800 0.95675000 -0.15046000

C 1.27006200 -0.30291700 -0.23431200

H 2.26008900 -0.66718900 -0.52924800

H -0.05948000 1.12693700 -0.09229600

C -1.21142200 -0.30746900 0.49145400

O 0.38204100 -1.16408600 -0.00742200

H -0.89314800 -0.31728800 1.53635400

H -1.89844400 -1.10229800 0.20374400

NH3

0 1

N 0.00000100 0.00000300 -0.11212000

H -0.90931200 -0.24453500 0.26161000

H 0.66642200 -0.66522900 0.26160900

H 0.24288700 0.90974100 0.26161800

[C6H5CHOO+NH3]

0 1

O -2.83650700 -1.40403600 -0.00062200

O -1.45797900 -1.31891300 -0.00022700

C -0.92942600 -0.16978600 -0.00079800

C 0.50387700 -0.03653200 -0.00036000

C 1.03951800 1.26108500 -0.00084200

C 2.41397200 1.44955700 -0.00045100

C 3.26498500 0.34872400 0.00040400

C 2.73993700 -0.94535000 0.00087600

C 1.37105800 -1.14377700 0.00050000

H -1.59635300 0.69133500 -0.00150900

H 0.37088500 2.11135700 -0.00150000

H 2.82075900 2.45089900 -0.00081400

H 4.33632100 0.49466900 0.00069800

H 3.40582300 -1.79690400 0.00154000

H 0.96023400 -2.14276900 0.00086000

N -3.73108500 1.57624500 0.00105400

H -4.24116200 1.90054500 -0.81239600

H -3.76697400 0.55680300 0.00102300

H -4.23958200 1.90040700 0.81553800

TS_NC1

0 1

O 3.50049400 0.10347400 0.33639100

O 2.10889600 -0.28845500 0.44362200

C 1.36688900 0.70127000 0.07238000

C -0.08108300 0.45621900 -0.03276200

C -0.95875900 1.54264200 -0.01033000

C -2.32973200 1.33865500 -0.10487000

C -2.83222100 0.04614000 -0.21945400

C -1.96116800 -1.04135500 -0.23900700

C -0.59072300 -0.84098200 -0.15023800

H 1.70081300 1.69217200 0.36279900

H -0.56642500 2.54654100 0.08890600

H -3.00389700 2.18354000 -0.08358300

H -3.89918400 -0.11457900 -0.29027200

H -2.35255900 -2.04575400 -0.32239200

H 0.09059000 -1.67939300 -0.15892600

N 2.12936900 0.94193500 -1.72530300

H 2.16052300 1.86851600 -2.12716000

H 3.03643700 0.65828900 -1.28389100

H 1.79010200 0.26338800 -2.39347100

HPMA

0 1

O -3.27617800 -0.24790800 -0.76188500

O -1.85665600 -0.52448500 -0.74159900

C -1.23700200 0.46986600 0.07567800

C 0.24842200 0.20017200 -0.01022400

C 1.13215200 1.24307100 -0.28172200

C 2.50457000 1.01725200 -0.31242300

C 3.00518100 -0.25893500 -0.07926000

C 2.12764000 -1.30781000 0.18126300

C 0.75686700 -1.08123400 0.21645000

H -1.47154100 1.45351900 -0.33484300

H 0.74628700 2.23620400 -0.47285800

H 3.17970100 1.83515100 -0.52382900

H 4.07135700 -0.43730200 -0.10646700

H 2.51103600 -2.30469200 0.35105100

H 0.07830000 -1.90271800 0.40060800

N -1.80796500 0.40045700 1.41654000

H -1.87167400 1.31224400 1.84675700

H -3.47835900 -0.30378500 0.18911300

H -1.27366300 -0.20696100 2.02398000

TS_ND1

0 1

O -3.44834300 1.14879000 -0.78303800

O -3.06014500 -0.31895000 -0.76801600

C -1.04363800 0.18623900 0.48002400

C 0.39188900 0.05374200 0.30221400

C 1.06132400 1.04674800 -0.44693100

C 2.44003400 0.97923600 -0.64596100

C 3.17361800 -0.08502100 -0.10564100

C 2.52059100 -1.08595800 0.62892700

C 1.14286900 -1.02109800 0.83009500

H -1.57055200 0.97827400 -0.03886600

H 0.48642000 1.86714100 -0.86486100

H 2.94239500 1.74986400 -1.22079700

H 4.24601900 -0.14026400 -0.26147600

H 3.08713200 -1.91777200 1.03414000

H 0.64800000 -1.81998100 1.37419600

N -1.75422900 -0.49672900 1.37255900

H -1.34930900 -1.18415800 1.99260800

H -3.68899800 1.27344200 -1.72680500

H -2.74217000 -0.55561000 1.06724600

C6H5CH=NH

0 1

C -0.77787400 0.01853500 0.10727300

C 0.70184800 -0.03559700 0.13492300

C 1.42293700 0.96237700 -0.54589600

C 2.82026300 0.95745400 -0.55161600

C 3.51730900 -0.04922700 0.12632400

C 2.81023600 -1.04930400 0.80881800

C 1.41481600 -1.04252000 0.81298400

H -1.18937500 0.86016300 -0.45565700

H 0.87903200 1.74272000 -1.07120000

H 3.36300400 1.73368600 -1.08129200

H 4.60254200 -0.05640000 0.12431000

H 3.34848700 -1.83086600 1.33541600

H 0.87849200 -1.82301200 1.34567800

N -1.64389100 -0.77069300 0.64219300

H -1.23190700 -1.55809800 1.16806400

[CH2OO+NH3]

0 1

O -0.84264100 -1.15912500 0.18528100

O -1.07557100 0.06935800 -0.38565400

C -0.66016200 1.07940000 0.22137400

H -0.83127300 2.02235300 -0.27953700

H -0.18560600 0.97356500 1.18514700

N 1.97773800 0.01896200 -0.04626700

H 1.35428800 -0.78399600 0.01384100

H 2.41317100 0.00454700 -0.96051300

H 2.71191700 -0.10746500 0.63966900

TS_NC2

0 1

O -0.99528300 -0.73739800 0.88035100

O 0.22093700 0.01475500 1.01295100

C 1.22379100 -0.75592200 0.99693600

H 2.18951000 -0.26391800 0.97670600

H 1.11630800 -1.76587100 1.36793700

N 0.97250400 -1.50180900 -0.94685700

H -0.03383700 -1.44781200 -0.73397900

H 1.21609000 -0.85146900 -1.68194200

H 1.23757600 -2.44241200 -1.20650500

HMA

0 1

O -1.43488400 -0.53223800 0.15617500

O -0.70613500 0.61534600 -0.33976700

C 0.57319900 0.60485500 0.27510500

H 0.99384900 1.55922600 -0.05188700

H 0.45808700 0.60129000 1.35964600

N 1.33615200 -0.55247700 -0.13072500

H -0.92357700 -1.25571200 -0.24152100

H 1.91041600 -0.39085800 -0.94511100

H 1.89712500 -0.94060600 0.61205000

TS_ND2

0 1

O -1.57231600 -0.97175800 -0.03544800

O -1.23916200 0.45306000 -0.45948800

C 1.09340400 0.62717000 0.46669600

H 0.59477400 1.48651400 0.05484400

H 1.63228100 0.68827600 1.40711700

N 1.07617200 -0.48895000 -0.21731000

H -2.34964900 -0.81825400 0.54450300

H 0.28177300 -0.49109000 -0.88926800

H 1.48450400 -1.35166100 0.11483500

CH2=NH

0 1

C 1.69821700 0.61653500 0.00984600

H 0.91413500 1.36912600 0.02599900

H 2.73685700 0.96004500 -0.00299500

N 1.34291900 -0.61585100 0.00545800

H 2.10592900 -1.30279600 -0.01270400
